# Quantification of hematopoietic stem and progenitor cells by targeted DNA methylation analysis

**DOI:** 10.1186/s13148-023-01521-w

**Published:** 2023-06-27

**Authors:** Ledio Bocova, Wouter Hubens, Cordula Engel, Steffen Koschmieder, Edgar Jost, Wolfgang Wagner

**Affiliations:** 1 Institute for Stem Cell Biology, University Hospital of RWTH Aachen, Aachen, Germany; 2 Helmholtz Institute for Biomedical Engineering, RWTH Aachen University, Aachen, Germany; 3 Department of Gynecology and Obstetrics, University Hospital of RWTH Aachen, Aachen, Germany; 4 Department of Hematology, Oncology, Hemostaseology and Stem Cell Transplantation, University Hospital of RWTH Aachen, Aachen, Germany; 5 Center for Integrated Oncology Aachen Bonn Cologne Düsseldorf (CIO ABCD), Aachen, Germany

**Keywords:** DNA methylation, Biomarker, Epigenetic, Hematopoietic stem cells, HSC, CD34, Blast, Leukemia

## Abstract

Hematopoietic stem and progenitor cells (HSPCs) are quantified in daily clinical practice by flow cytometry. In this study, we provide proof of concept that HSPCs can also be estimated by targeted DNA methylation (DNAm) analysis. The DNAm levels at three individual CG dinucleotides (CpG sites) in the genes *MYO1D*, *STK17A*, and *SP140* correlated with CD34^+^ cell numbers in mobilized peripheral blood and with blast counts in leukemia. In the future, such epigenetic biomarkers can support the evaluation of stem cell mobilization, HSPC harvesting, or blast count in leukemia.

**Supplementary Information:**

The online version contains supplementary material available at 10.1186/s13148-023-01521-w.

## Introduction

Monitoring of hematopoietic stem and progenitor cells (HSPCs) is crucial to determine the efficiency of HSPC mobilization for stem cell apheresis in clinical routine. These cells are usually determined by flow cytometry based on the cell surface marker CD34 [1]. For more detailed quantification of early hematopoietic progenitors, such as primitive hematopoietic stem cells (HSCs), lymphoid-primed multipotent progenitors (LMPPs), or common myeloid progenitors (CMPs), additional surface markers and the absence of lineage-specific markers can be utilized [2]. However, immunophenotypic analysis necessitates fresh blood samples, and measuring of multiple surface markers is labor-intensive and costly.

The composition of cells in tissue can also be estimated based on epigenetic parameters [3]. DNA methylation (DNAm) is a reversible modification of cytosine residues particularly at CG dinucleotides (CpG sites). Epigenetic signatures based on hundreds of CpGs have been used for deconvolution of leukocyte subsets [4]. The first predictors were generated and applied on Illumina Bead Chip Microarray datasets—initially on the 450k platform [4] and more recently on the human EPIC 850k Bead Chip [5]. Furthermore, multi-CpG signatures have been adjusted to better discern additional and non-hematopoietic cell types [6]. We have previously demonstrated that even targeted analysis of individual cell-type specific CpG sites can be used to estimate granulocytes, CD4 T cells, CD8 T cells, B cells, NK cells, and monocytes [7, 8]. However, so far epigenetic biomarkers have not been established for HSPCs. We have therefore revisited DNAm profiles of sorted subsets from peripheral blood to identify CpG sites that might provide reliable biomarkers for HSPCs.

## Methods

### Selection of cell-type specific CpGs

To identify CpG sites specific for HSPCs, HSCs, LMPPs, and CMPs, we compared 450k Illumina Bead Chip Microarray data from sorted cell types, available on Gene Expression Omnibus (GEO). A detailed description of the datasets and the analysis is provided in Additional file 1: Methods. In short, raw data processed with R. DNAm (*β*-values) were normalized with ssNoob and the candidate CpGs were identified based on two parameters as described before [7, 8]: (1) high difference in mean DNAm levels (*β*-values) between HSPCs *versus* all other cell types and (2) low variation of *β*-values within each of these two groups. We arbitrarily selected the most relevant CpGs according to both parameters, taking corresponding gene functions into account as well. DNAm of candidate CpGs was further investigated on 301 independent DNAm profiles of 42 different studies (Additional file 1: Table S1).

### Blood samples

Peripheral blood samples from healthy donors (PB, *n* = 8), PB from patients with hematologic diseases (*n* = 39; particularly acute myeloid leukemia), cord blood (CB, *n* = 5), mobilized peripheral blood (mPB, *n* = 9), and stem cell apheresis product (*n* = 6) were collected after informed and written consent according to guidelines specifically approved by the local ethics committee of the RWTH Aachen University (EK206/09, EK099/14).

### Pyrosequencing

Genomic DNA was isolated using QIAamp DNA Mini Kit (Qiagen) or NucleoSpin Tissue XS (Macherey Nagel) and subsequently bisulfite-converted using EZ DNA Methylation Kit (Zymo research). Bisulfite-converted DNA (10–20 ng) was amplified using PyroMark PCR Kit (Qiagen) with primers designed with PyroMark Assay Design 2.0 software (Qiagen) and purchased at Metabion (Additional file 1: Table S2). PCR amplicons were sequenced on a PyroMark Q96 ID (Qiagen), and all measurements are provided in Additional file 2: Table S3.

Further information on CD34^+^ cell sorting, colony forming unit assays, DNA isolation, pyrosequencing, models for the cellular deconvolution, and gene expression analysis is provided in Additional file 1: Methods.

## Results and discussion

**Fig. 1:**
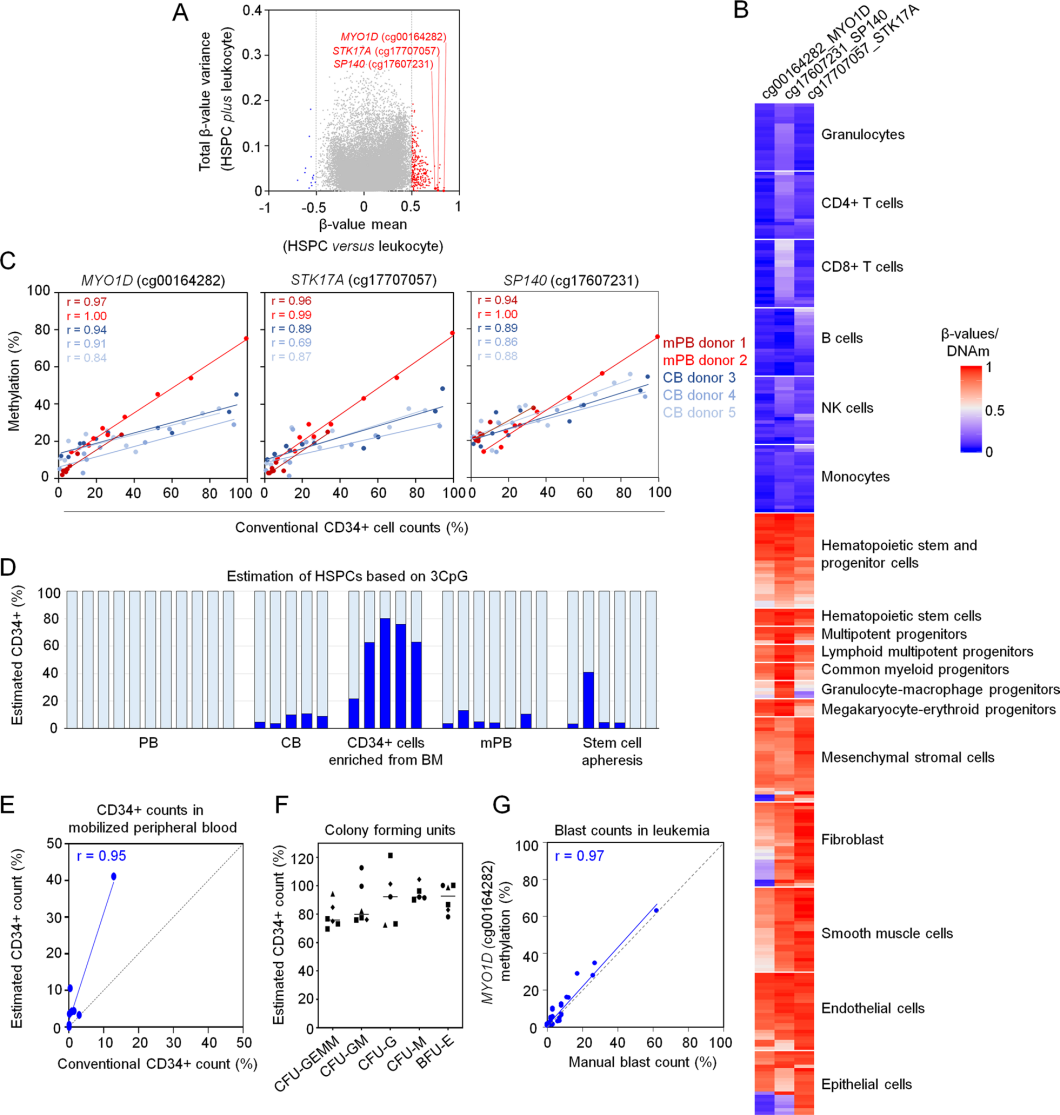
Epigenetic biomarker for hematopoietic stem and progenitor cells. **A** Candidate CpG sites to discriminate HSPCs (GSE72867) from other leukocytes (GSE35069) were selected based on the difference in mean DNAm levels (*β*-value) and variation of *β*-values within these two groups. **B** DNAm levels of the three candidate CpGs are depicted in independent DNAm profiles (*n* = 301) of 42 studies (Additional file 1: Table S1). **C** DNAm levels at the three relevant CpGs in the genes *MYO1D*, *STK17A*, and *SP140* were analyzed with pyrosequencing in dilutions of CD34^+^ HSPCs from mobilized peripheral blood (mPB, *n* = 2) and cord blood (CB, *n* = 3), measured with flow cytometry. **D** Multivariable models for the three CpGs were trained on the dilution data for mPB or CB. These models were then applied to estimate the fraction of HSPCs in different types of frozen samples (mPB model for PB, mPB, CD34^+^ BM cells, and apheresis samples; CB model for CB). **E** Estimates of HSPC counts based on the mPB multivariable model were compared to flow cytometric CD34 measurements in mPB (*n* = 9). **F** The CB HSPC predictor was applied to individual colonies in colony forming units (CFUs). **G** Correlation of DNAm at the CpG in *MYO1D* (cg00164282) with manual counts of blasts in leukemic samples (*n* = 39). Correlations were assessed by Pearson correlation coefficient r

To select individual CpGs that discriminate between HSPCs and other cell types, we used profiles of CD34^+^ cells from mobilized peripheral blood (mPB) and of purified leukocyte subsets. Cell-type specific CpGs were identified as described before [7, 8], and most of these were hypermethylated in HSPCs. We selected three candidate CpGs that were located within the genes serine/threonine kinase 17a (*STK17A*, cg17707057), myosin ID (*MYO1D*, cg00164282), and SP140 nuclear body protein (SP140; cg17607231) (Fig. 1A). These CpGs were either localized in the gene body (*SKT17A, MYO1D*) or in the promoter region of corresponding genes (*SP140*; Additional file 1: Fig. S1). To further validate that the CpGs can discern HSPCs from other cell types, we compiled a dataset of 301 DNAm profiles (Additional file 1: Table S1). In fact, all three CpGs were consistently methylated across various subsets of HSPCs, while they were hypomethylated in mature leukocytes of all lineages. Notably, the HSPC-associated CpGs were also methylated in other non-hematopoietic cell types, such as fibroblasts, endothelial cells, and epithelial cells (Fig. 1B). Thus, the three candidate CpGs are not specifically methylated in HSPCs, but rather specifically hypomethylated during hematopoietic differentiation.

To assess whether these three CpG sites can be used for targeted deconvolution of HSPC fractions, we created artificial mixtures of CD34^+^ and CD34^−^ cells, derived from cord blood (CB, *n* = 2), and mobilized peripheral blood (mPB; *n* = 3). Pyrosequencing essays were established and tested on DNA isolated from these artificial mixes. Overall, the DNAm levels at the three CpGs were higher in CD34^+^ cells from mPB as compared to CB, which might be attributed to epigenetic differences between fetal and adult hematopoiesis (Fig. 1C). Despite this difference, DNAm levels in dilutions of all donor samples revealed a high correlation with CD34^+^ counts determined by flow cytometric measurements, further substantiating that the three candidate CpGs can be indicative for HSPC fractions.

To estimate HSPC fractions, we trained a three CpG multivariable model based on the dilution measurements for either mPB or CB. The CB model was initially tested on cryopreserved cord blood samples (*n* = 5), and the mPB model was tested on peripheral blood (*n* = 11), CD34^+^-enriched cells from bone marrow (*n* = 5), mobilized peripheral blood (*n* = 7), and stem cell apheresis products (*n* = 6; Fig. 1D). As expected, HSPCs were not predicted in non-mobilized blood, and the highest number of HSPCs was estimated in CD34^+^-enriched cells from bone marrow. Furthermore, predictions correlated with flow cytometric analysis of CD34^+^ counts in mPB (*n* = 9; *r* = 0.95, albeit the HSPC numbers were overestimated and correlation is particularly driven by one leverage point (Fig. 1E)). We have also tested our HSPC predictor on colony forming units (CFUs) derived from clonogenic HSPCs. CFUs consistently revealed high predictions for HSPCs (Fig. 1F). This might be expected, given that CFUs are used as surrogate assay for HSPCs. Next, we tested whether the HSPC predictor might also reflect leukemic blast counts, which also often express the CD34 antigen and derive from HSPC-related cell types. We found that particularly the DNAm levels at *MYO1D* showed a high correlation with blast counts (*n* = 39; *r* = 0.97; Fig. 1G). Blast counts also correlated with DNAm at *STK17A* (*r* = 0.92) and to a lesser extent at *SP140* (*r* = 0.62; Additional file 1: Fig. S2A). Yet, our predictors for HSPCs in blood revealed offsets for estimating absolute blast counts (Additional file 1: Fig. S2B). Either way, these results indicate that such epigenetic analysis may also support the evaluation of blast counts, especially if driver mutations are not available to determine blast burden more precisely.

**Fig. 2:**
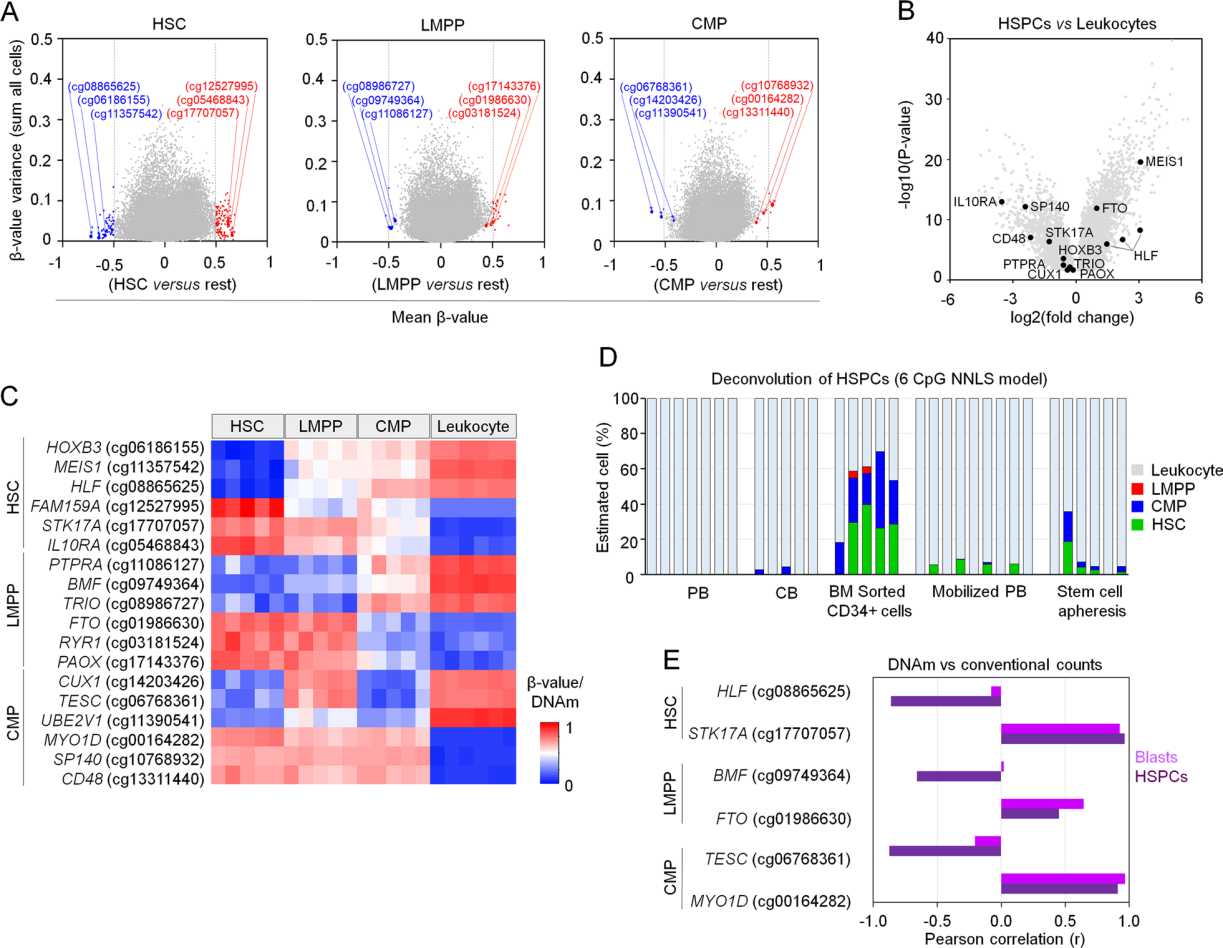
DNA methylation in subsets of hematopoietic progenitor cells. **A** Candidate CpGs were selected for hematopoietic stem cells (HSCs), lymphoid-primed multipotent progenitor (LMPPs), and common myeloid progenitor cells (CMPs). The selection is based on (1) difference of mean *β*-value in DNAm profiles of these progenitor subsets (GSE63409) in comparison with all other subsets and leukocytes (GSE35069) and (2) variance of *β*-values within these groups. Three hypo- (blue) and three hypermethylated CpGs (red) were depicted for each subset. **B** Gene expression profiles were compared in HSPCs versus leukocytes (GSE24759), and 12 of the 18 selected CpGs were significantly differentially expressed (adjusted *P* value < 0.05). **C** Heatmap depicts DNAm (*β*-values) for 18 CpGs (GSE63409, GSE35069). **D** Estimation of HSPC subsets based on a non-negative least square model (NNLS model) for 6 CpGs in different types of cryopreserved blood samples. **E** Pearson correlation of DNAm levels in pyrosequencing with CD34 counts in mPB (HSPCs, flow cytometry, *n* = 9) and leukemic blasts (manual counts, *n* = 39)

There are lineage-specific DNAm differences between the small fraction of hematopoietic stem cells, myeloid progenitors, and lymphoid progenitors [2, 9]. Thus, we investigated whether even subsets of HSPCs might be discerned based on DNAm of individual CpGs. To this end, we used 450k DNAm profiles of CMPs, LMPPs, and HSCs (Additional file 1: Fig. S3) [2]. For each of these subsets, we selected three hypo- and three hyper-methylated candidate CpGs in comparison with the other two HSPC subsets and leukocyte subsets (Fig. 2A). *MYO1D* and *STK17A* were again within the top 18 candidate CpGs (Additional file 1: Table S4 and Additional file 3: Table S5). Notably, many of the corresponding genes have previously been shown to be higher expressed in primitive hematopoietic stem cells, including *HOXB3*, *MEIS1*, *CD48*, and hepatic leukemia factor (*HLF)* [10]. In fact, HLF seems to be a key regulator of earliest lineage commitment at the transition from multipotency to lineage-restricted progeny [11]. When we analyzed differential gene expression in HSPCs *versus* leukocytes (GSE24759) we found that 12 of the 18 genes were significantly differentially expressed (adjusted *P* value < 0.05; Fig. 2B). Thus, the selected candidate CpGs overall seem to be related to functionally relevant genes. DNAm levels of all 18 CpGs were able to discern hematopoietic progenitor cells from leukocytes (Fig. 2C). We also observed clear DNAm differences between LMPPs and CMPs. Yet, they were not clearly demarcated from DNAm patterns of HSCs. This suggests that hematopoietic differentiation is a continuum with no clearly defined intermediate states. Furthermore, in contrast to the CpGs initially selected for CD34^+^-associated cells, the DNAm levels varied greatly in non-hematopoietic cell types (Additional file 1: Fig. S4).

Despite this limitation, we tried to estimate the composition of HSPCs based on targeted DNAm analysis with pyrosequencing. In order to reduce the labor-intensive work and costly analysis of all 18 CpG sites, we focused on six CpGs (one hypo- and one hypermethylated CpG for each HSPC subtype): *HLF* (cg08865625), *STK17A* (cg17707057), Bcl-2-modifying factor (*BMF*, cg09749364), FTO alpha-ketoglutarate-dependent dioxygenase (*FTO*, cg01986630), tescalcin (*TESC*, involved in myeloid differentiation, cg06768361), and *MYO1D* (cg00164282). For epigenetic predictions, a non-negative least square model (NNLS model) was trained on the mean DNAm data of the reference datasets for HSCs, LMPPs, CMPs, and leukocytes (Additional file 4: Table S6) [8]. As there were no available DNAm profiles with known composition of the different HSPC subsets, we applied our NNLS model on the same PB, CB, BM-derived sorted CD34^+^ cells, mPB, and stem cell apheresis samples that we used for estimating total HSPCs (Fig. 2D). The sum of the estimated HSCs, LMPPs, and CMPs fractions was very similar to the total HSPC fraction we predicted with our multivariable HSPC model (Fig. 1D). When we tested this approach on individual CFUs, particularly CFU-GEMM and BFU-E were predicted to have higher fractions of progenitor cells (Additional file 1: Fig. S5). All of the six selected CpGs correlated with CD34^+^ counts in mPB (*n* = 9). Furthermore, all three hypermethylated CpGs had very high correlation with blast counts in leukemia samples (n = 39; *STK17A* r = 0.92; *FTO* r = 0.64; *MYOD1* r = 0.96), whereas this was not observed for the hypomethylated CpGs (Fig. 2E). It is well known that aberrant DNAm exists in leukemia that varies extensively between different samples [12]. However, the correlation of our hypermethylated candidate CpGs with blast counts indicates that DNAm at these CpGs is affected to a lesser degree by disease entity or patient-specific variation.

Taken together, our study provides proof of principle that epigenetic measurements can reflect the fraction of HSPCs in blood. This approach may facilitate monitoring of hematopoietic stem cell mobilization or measuring of HSPCs in a transplant. In contrast to flow cytometric measurements, DNAm analysis is also applicable to frozen blood or dried blood spots, enabling retrospective analysis or self-assessment with a finger prick [7, 8]. The epigenetic biomarkers might even track numbers of leukemic blasts. While the high correlations of our results with CD34 counts or blast counts are promising, further validation in larger cohorts is needed. Particularly for blast cells, which may vary extensively in their epigenetic makeup between different disease entities, larger cohorts should be considered that include specific types of leukemia. We also like to note that a limitation of our study—and of epigenetic deconvolution in general—is that, when analyzing bulk DNA, it may not be possible to reliably discern subsets that are present in very low quantities, such as HSPCs. Even when CpG sites have been identified with very high methylation differences in the cell type of interest, this will barely affect the overall methylation values of the bulk DNA. To tackle this, methods with very high precision and accuracy are required. In principle, multi-CpG signatures for Illumina Bead Chip data can provide additional controls for bisulfite conversion, better correction for SNPs, and larger signatures may be more redundant and thus more stable. However, these microarrays can hardly be used for clinical diagnostics since they are not accredited for diagnostic application [13]. For clinical applications, site-specific DNAm analysis might therefore be more advantageous and the precision of this approach might be further improved in the future—for instance, by using digital droplet PCR (ddPCR) instead of pyrosequencing. Notably, several ddPCR machines are already approved for clinical application, e.g., in Europe under the in vitro diagnostic medical device directive (IVDD) [13]. While it is currently unlikely that epigenetic quantification of HSPCs will replace the conventional methods, it could be useful to confirm measurements for stem cell mobilization, quality control of apheresis samples, and to estimate leukemic blasts in the future.

## Supplementary Information

**Additional file 1.** Supplemental methods, supplemental figures, and supplemental tables S1, S2, and S4.

**Additional file 2.** **Table S3.** DNA methylation results of pyrosequencing measurements.

**Additional file 3.** **Table S5.** Table with calculation of mean beta-values and variances for selection of candidate CpGs.

**Additional file 4.** **Table S6.** Application for NNLS-model with 6 CpGs.

## Data Availability

The DNAm and gene expression datasets analyzed in this study are available in NCBI´s Gene Expression Omnibus repository (https://www.ncbi.nlm.nih.gov/geo/) under the accession numbers as indicated in the text.
