# Correction: Quantification of hematopoietic stem and progenitor cells by targeted DNA methylation analysis

**DOI:** 10.1186/s13148-023-01532-7

**Published:** 2023-07-20

**Authors:** Ledio Bocova, Wouter Hubens, Cordula Engel, Steffen Koschmieder, Edgar Jost, Wolfgang Wagner

**Affiliations:** 1 Institute for Stem Cell Biology, University Hospital of RWTH Aachen, Aachen, Germany; 2 Helmholtz Institute for Biomedical Engineering, RWTH Aachen University, Aachen, Germany; 3 Department of Gynecology and Obstetrics, University Hospital of RWTH Aachen, Aachen, Germany; 4 Department of Hematology, Oncology, Hemostaseology and Stem Cell Transplantation, University Hospital of RWTH Aachen, Aachen, Germany; 5 Center for Integrated Oncology, Aachen Bonn Cologne Düsseldorf (CIO ABCD), Aachen, Germany

**Correction: Clinical Epigenetics (2023) 15:105** 10.1186/s13148-023-01521-w

The wrong Additional file 4 was originally published with this article [1]. It has now been replaced with the correct file (Additional file 4).

The original article has been corrected.

## Supplementary Information

**Additional file 4. Table S6.** Application for NNLS model with 6 CpGs.
