# Supplementary material for: Quantification of hematopoietic stem and progenitor cells by targeted DNA methylation analysis
Source: Clin Epigenetics. 2023 Jun 27;15:105. doi: 10.1186/s13148-023-01521-w (PMC10303729; doi:10.1186/s13148-023-01521-w)
Supplement: Supplementary file 1 — Additional file 1. Supplemental methods, supplemental figures, and supplemental tables S1, S2, and S4. [file 13148_2023_1521_MOESM1_ESM.pdf]

Supplementary Materials

Quantification of hematopoietic stem and progenitor cells by targeted DNA methylation analysis

Ledio Bocova, Wouter Hubens, Cordula Engel, Steffen Koschmieder, Edgar Jost, and Wolfgang Wagner

Supplemental methods

Additional information for analysis of DNAm profiles.

For the selection of candidate CpGs for HSPCs, we used Illumina 450k BeadChip datasets of sorted CD34+ cells from five donors, each with three technical replicas (GSE72867) (1). Their DNAm profiles were compared to other sorted leukocyte subsets (granulocytes, monocytes, CD4 T cells, CD8 T cells, NK cells, B cells) from 6 donors (GSE35069) (2). The later dataset was also used for our previous selection of cell-type specific CpGs (3, 4). In analogy, we also selected CpGs for LMPPs, CMPs, and HSCs by analyzing DNAm profiles of these cell subsets isolated from 5 normal bone marrow samples (GSE63409) (5). The raw IDAT files were retrieved from Gene Expression Omnibus (GEO) and the data was processed using minfi in R (version 4.1.3) (6). Features were limited to CpGs shared between the 450K and EPIC platforms, and we excluded bad quality probes (P-value > 0.05), probes related to sexual chromosomes, and probes not shared across all samples. Furthermore, the DNAm (β-values) were normalized using the single-sample Noob (ssNoob) application (7). Noob (normal-exponential out-of-band) is a background correction method with dye-bias normalization for Illumina Infinium methylation arrays, and this normalization procedure was further developed for incremental preprocessing of individual methylation arrays for cross-study comparison (extended minfi package) (7). The selection of CpGs was based on mean DNAm differences and variation within the groups. This selection was based on an Excel Table (Supplemental Table S5) that was also used for the graphical presentation. While the selection of relevant CpGs in this study was not based on a specific R code, we have meanwhile further developed this approach into CimpleG (https://github.com/CostaLab/CimpleG) (8). For graphical presentation, CpGs were classified as hypermethylated (red) or hypomethylated (blue) in HSPCs, using cutoffs of difference of mean β-value >0.5, or <-0.5, respectively. Heatmaps were generated with heatmapper (9). Pearson correlation (r) between DNAm and age was calculated for each individual CpG.

Additional information for pyrosequencing

Specific regions of the bisulfite treated DNA were amplified in a thermocycler (Eppendorf) using the following program: DNA polymerase activation for 15 min at 95°C; 45 cycles of DNA denaturation (30 seconds at 94°C), annealing (30 seconds at 56°C) and DNA extension (30 seconds at 72°C); and at the end extension for 10 minutes at 72°C. Subsequently, 20 µl PCR products were bound to 5 µl Streptavidin Sepharose High Performance Beads (GE Healthcare) and annealed to 1 µl sequencing primers (5 µM) for 2 min at 80°C. PCR amplicons were sequenced on a PyroMark Q96 ID (Qiagen) and analyzed with PyroMark Q96 CpG 1.0.9 (Qiagen).

CD34+ cell sorting and cell dilutions

Mononuclear cells were isolated from cord blood or mobilized peripheral blood (10-20 ml) using Ficoll density gradient centrifugation. Next, $CD34^{+}$ cells were separated using magnetic-activated cell sorting (MACS) with the CD34 MicroBead Kit (Miltenyi). The purified cells were quantified manually with Neubauer chamber and mixed in different $CD34^{+}$ cell proportions with the $CD34^{-}$ flow-through (0-100%; mixtures of

cells, not of DNA). To determine the CD34-content of these dilutions, cells were stained with APC-anti CD34 and V500-anti CD45 antibodies. Fluorescence intensities were measured with FACS Canto II Becton Dickinson (BD) and the data was acquired using BD FACSDiva™ software and analyzed with FlowJoTM v10.

Analysis of colony forming units

Colony forming units (CFUs) arise from primitive clonogenic HSPCs. To estimate if this was also reflected in our epigenetic biomarkers, we generated CFUs from the cord blood of four donors. About 1000 $CD34^+$ cells were cultured in human StemMACS™ HSC-CFU Media with EPO (Miltenyi), supplemented with cytokines for myeloid differentiation (GM-CSF, IL-3, Erythropoietin). After 14 days incubation at 37°C the colonies were scored by morphological parameters according to the manufacturer's instructions into the following types of colonies: CFU-E = CFU erythrocyte; BFU-E = burst forming unit erythrocyte; CFU-M = CFU macrophage; CFU-G = CFU granulocyte; CFU-GM = CFU granulocyte-macrophage; CFU-GEMM = CFU granulocyte-erythrocyte-macrophage-megakaryocyte. Individual colonies were picked with a pipet tip for DNA isolation using a Leica DM1000 LED microscope (Leica Microsystems) at 40X magnification.

DNA isolation

DNA was isolated from blood samples using QIAamp DNA Mini Kit (Qiagen). In short, cells were lysed for 10 min at 56°C with proteinase K. Silica gel solid support spin columns were used to bind and clean the DNA from lysed samples. DNA was eluted in 150 µl elution buffer and the concentration was measured by Nanodrop (Thermo Scientific) and stored in -20°C freezer until further analysis. For samples with lower cell numbers such as individual blood colony forming units or artificial cell dilutions, DNA was isolated with NucleoSpin Tissue XS (Macherey Nagel). Cells were lysed for 15 min at 70°C with proteinase K in sodium dodecyl sulfate (SDS) lysis buffer and silica gel solid support spin columns were used to bind and clean the DNA. DNA was eluted in 20 µl elution buffer (5 mM Tris/HCl, pH 8.5).

Models for epigenetic estimation of HSPCs

To derive an epigenetic predictor for HSPC content, we trained a multivariable model based on the artificial dilution results of five donors (in total 47 measurements; Figure 1C). Since there was a DNAm difference between CB and mPB CD34 cell dilutions, we trained two alternative models for these specimens with Excel:

$$HSPCs^{CB} = 0.6232*DNAm^{cg17707057} + 1.236*DNAm^{cg00164282} + 0.95*DNAm^{cg17607231} - 34.38$$

$$HSPCs^{mPB} = 0.444*DNAm^{cg17707057} +0.3123*DNAm^{cg00164282} +0.6601*DNAm^{cg17607231} -13.070$$

For deconvolution of the HSPC subsets we use a non-negative least square model (NNLS model) as described before (3). This model was based on the matrix of mean DNAm levels of the reference datasets (supplemental table S4). For deconvolution of HSPC subsets in CFU data, we focused on the following six CpGs, since the amount of DNA for individual colonies was very low: *BMF* cg09749364; *FTO* cg01986630; *HLF* cg08865625; *STK17A* cg17707057; *TESC* cg06768361; and *MYO1D* cg00164282. An application for cell type deconvolution is provided as a separate Excel tool for the 6 CpG NNLS-model (Supplemental Table S6).

Gene expression analysis

To estimate whether DNAm at candidate CpGs is also reflected on gene expression level, we used microarray data of purified hematopoietic cell subsets from umbilical cord blood (GSE24759)(10) and compared expression levels between HSPCs (n=34) and mature leukocytes (n=118). Data were analyzed with GEO2R and normalized with quantile normalization using limma R package (version 3.54.0) (11). Significant genes (adjusted P-value <0.05) were selected and P-value was plotted against the log2 fold change.

Supplemental figures

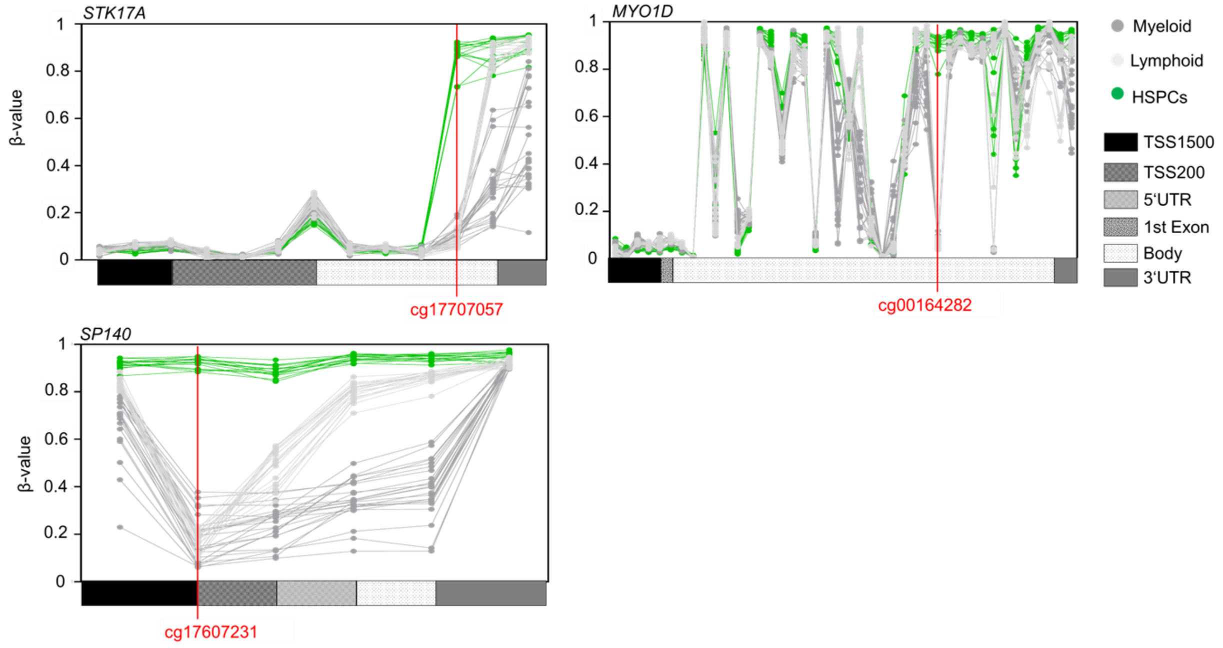

Supplementary Figure S1. DNA methylation in genes with candidate CpGs.

The candidate CpGs for hematopoietic stem and progenitor cells (HSPCs, GSE72867) were identified in the genes *MYO1D*, *STK17A*, *SP140.* This figure depicts DNAm levels (β-values) of the different probe sets on the 450k Illumina BeadChip for these genes in relation to different parts of the genes (TSS1500 = 1500bp upstream of transcripton start site, TSS200, 5'UTR = 3'untranslated region, 1st Exon, Body, and 3'UTR, according to the Illumina annotation). For comparison DNAm levels are predicted for myeloid and lymphoid mature cells (GSE35069).

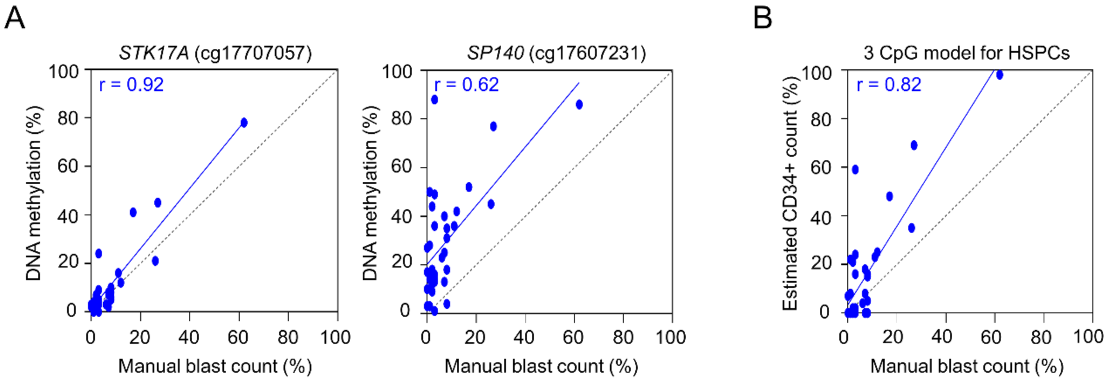

Supplementary Figure S2. HSPC predictors and DNA methylation in genes with candidate CpGs.

A) Correlation of DNAm at the CpGs in *STK17A* (cg17707057) and in *SP140* (cg17607231) with manual counts of blasts in leukemic samples (n=39). **B)** Estimates of HSPC counts based on the mPB multivariable model were compared to manual counts of blasts in leukemic samples (n=39). Pearson correlation coefficients (r) are provided.

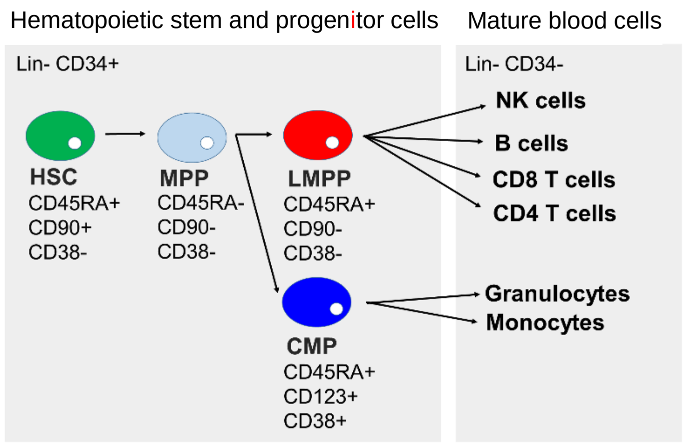

Supplementary Figure S3. Schematic presentation of hematopoietic subsets.

The schematic presentation depicts the surface markers that were used to sort the HSPC subsets for CMPs, LMPPs, and HSCs (5).

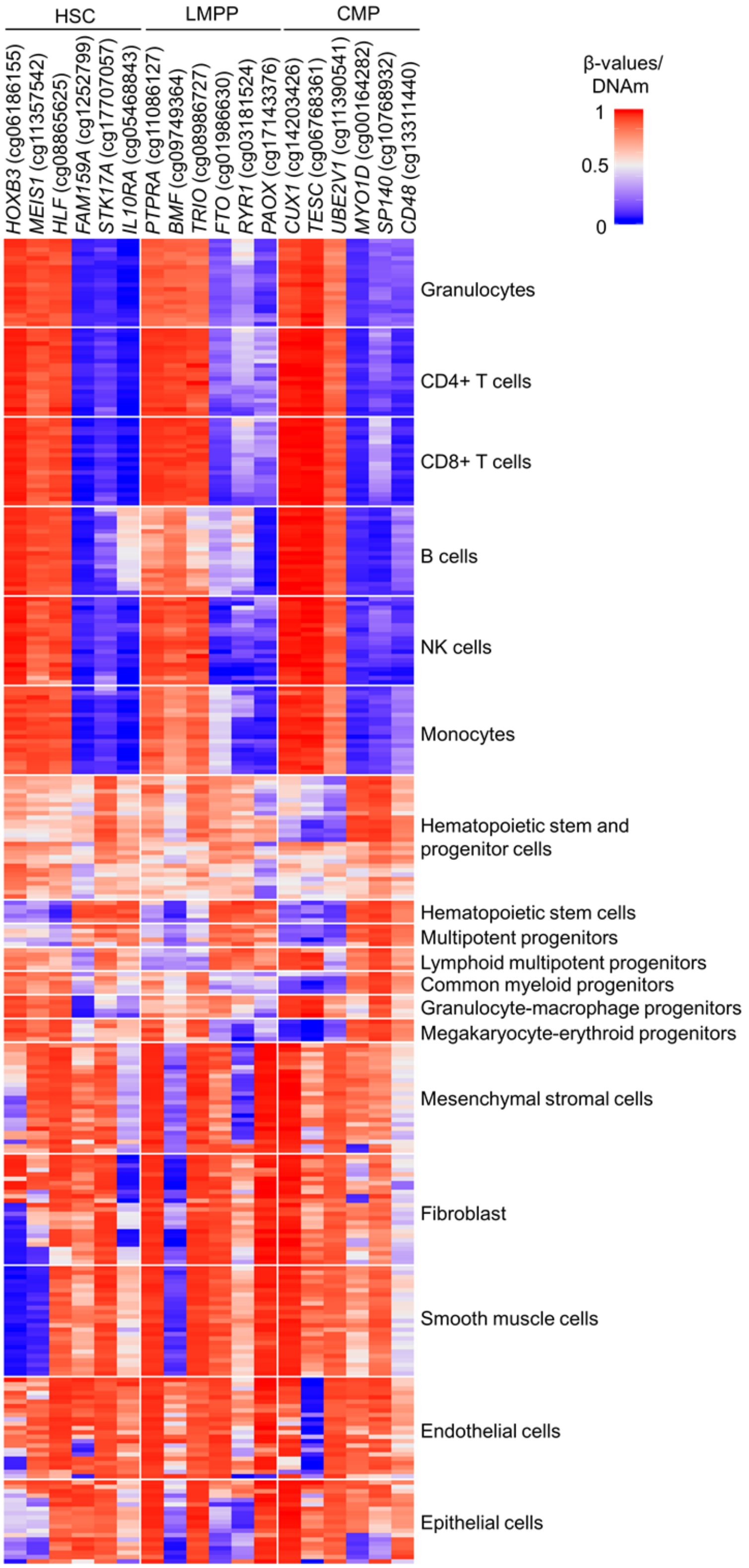

Supplementary Figure S4. DNA methylation levels at candidate CpGs in different cell types.

The DNA methylation levels (β-values) are depicted for the 18 candidate CpG sites for the subsets of HSPCs in various cell types. The heatmap depicts the ssNoob normalized data of 301 cell preparations that derived from 42 different studies (supplemental table S1).

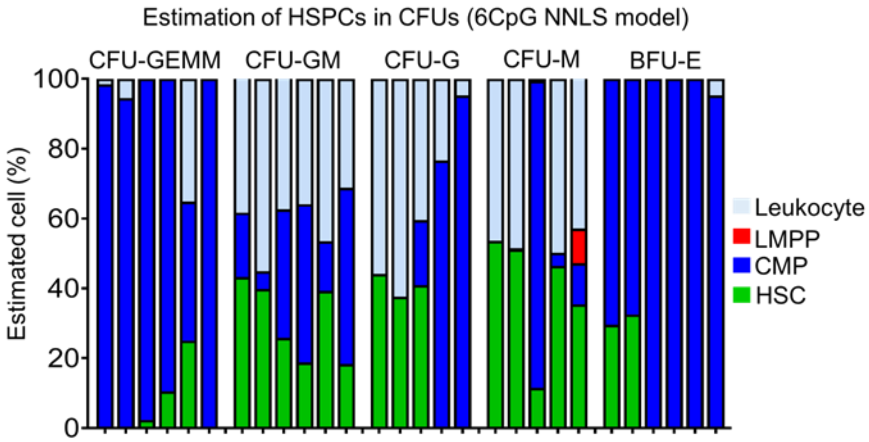

Supplementary Figure S5. Predictions of hematopoietic progenitor cells in colony forming units.

CD34+ cells from cord blood were seeded in colony forming unit assays and after 14 days of culture individual colonies were picked for DNA isolation. The DNA was bisulfite converted and analyzed by pyrosequencing at six CpGs (one hyper and one hypomethylated CpG for HSCs, LMPPs, and CMPs). We could not address all 18 CpGs, because the amount of DNA is very small. The DNAm levels were used for a non-negative least square (NNLS) model to predict the composition of HSPC subsets. For all colonies, high numbers of progenitor cells were predicted – particularly for CFU-GEMM and BFU-E, which may be enriched in more primitive progenitor cells. On the other hand, CFU-GEMM and BFU-E were predicted to comprise lower HSC numbers, which may be counterintuitive given that particularly CFU-GEMM have high replating and self-renewal potential (12). It is conceivable, that the high proliferation rates in these colonies contribute to the low predictions of HSCs factions in these large colonies, but this needs to be further investigated in the future. Lymphoid progenitor cells were hardly predicted in all types of colonies, which is in line with the fact that CFU conditions favor myeloid lineage.

Supplemental Tables

Supplemental Table S1. DNA methylation studies for the reference dataset

GEO ID	Sample Type	Number of Samples
GSE35069	Granulocytes	6
GSE35069	CD4 T cells	6
GSE35069	CD8 T cells	6
GSE35069	Monocytes	6
GSE35069	B cells	6
GSE35069	NK cells	6
GSE40699	Epithelial cells	5
GSE49618	Hematopoietic stem and progenitor cells	3
GSE52025	Fibroblast	2
GSE58477	Hematopoietic stem and progenitor cells	10
GSE59065	CD4 T cells	4
GSE63409	Hematopoietic stem cells	6
GSE63409	Multipotent progenitor cells	6
GSE63409	Lymphoid multi progenitor cells	6
GSE63409	Common myeloid progenitors	6
GSE63409	Granulocyte-macrophage progenitors	6
GSE63409	Macrophage-erythroid progenitors	6
GSE65078	Fibroblast	4
GSE66562	NK cells	14
GSE72867	Hematopoietic stem and progenitor cells	15
GSE74877	Mesenchymal stromal cells	2
GSE74877	Fibroblast	2
GSE74877	Epithelial cells	1
GSE77135	Fibroblast	13
GSE79695	Mesenchymal stromal cells	12
GSE85566	Epithelial cells	6
GSE85647	Monocytes	4
GSE87797	Mesenchymal stem cells	6
GSE92843	Epithelial cells	1
GSE103541	Granulocytes	14
GSE106099	Endothelial cells	12
GSE107226	Fibroblast	4
GSE109042	Epithelial cells	6
GSE129266	Mesenchymal stromal cells	3
GSE130030	CD8 T cells	14
GSE130030	CD4 T cells	11
GSE131989	Monocytes	10
GSE131989	B cells	14
GSE140078	Endothelial cells	2
GSE140295	Endothelial cells	8
GSE146376	Smooth muscle cells	25
GSE196046	Mesenchymal stromal cells	2

Supplemental Table S2. Pyrosequencing and PCR primers

Primer	Sequence 5'-3'
BMF	
Forward	AGT TTG ATA GTA GGA TTT GGG ATA A
Reverse	Biotin-ACA ATA AAC CAT AAA TAA TCC CTC TTA AA
Sequencing	GGT TTT AGT TAG TAG GAG AG
FTO	
Forward	Biotin-TAG GAG GGT TGT GAG TTA GAA GTT
Reverse	ACA AAC CCC TTT AAT ATA ATA TTC AT
Sequencing	AAA TTA ACA CAT AAA ACC ACA
HLF	
Forward	Biotin-ATG GGG GAG TTG GTT AAA ATG TAT A
Reverse	ACT CCT AAC AAA AAC TCT CCC ACT TTA A
Sequencing	ACA TCC ATA TCA CTC ATC
STK17A	
Forward	TGA GTG GTT TGA AGG GTT TTT T
Reverse	Biotin-AAT TTA CTA ACC CCT ACT CTA TCC
Sequencing	AAG GTA TGT ATG TTT TTT TAA ATT A
TESC	
Forward	GGG AGA GGT TAG GGA AAT ATA GG
Reverse	Biotin-TAA CCC TAC CCT CCC CAA TA
Sequencing	GGA AAT ATA GGG TTA GTT TG
SP140	
Forward	AAG GGA GGA GGA GTA GAG TT
Reverse	Biotin-ATT TAC TTC CTC CTC ATA ATA CCA CCT TAC
Sequencing	GAG GAG TAG AGT TAG TTT T
MYO1D	
Forward	GGT TTG ATA TTA GTA GGG GGA TT
Reverse	Biotin-AAC CTT ACA CCA AAC AAA AAC AAA TAC TT
Sequencing	AGT GAT ATT TAG ATG GAG GA

Supplemental Table S3. DNA methylation results of pyrosequencing measurements

The results of the pyrosequencing measurements are provided as separate Excel table. The different pages of this file provide the data for Figures 1C, 1D, 2D, 1G&2E, S4, and 1E.

Supplemental Table S4. Average DNAm at the 18 selected CpGs in different cell types.

Gene ID	CpG ID	HSC AV	MPP AV	CMP AV	GMP AV	LMPP AV	MEP AV	Gran AV	CD4T AV	CD8T AV	Monoc AV	B cell AV	NK cell AV	Neu	Eosinophil
PTPRA	cg11086127	0.31	0.43	0.63	0.63	0.30	0.82	0.84	0.94	0.93	0.83	0.76	0.90	0.82	0.69
BMF	cg09749364	0.10	0.22	0.47	0.57	0.29	0.54	0.83	0.91	0.90	0.73	0.83	0.83	0.82	0.78
TRIO	cg08986727	0.37	0.41	0.73	0.61	0.32	0.77	0.81	0.94	0.93	0.82	0.66	0.93	0.84	0.92
FTO	cg01986630	0.79	0.73	0.36	0.71	0.76	0.29	0.18	0.23	0.11	0.45	0.37	0.12	0.17	0.06
RYR1	cg03181524	0.82	0.75	0.36	0.55	0.77	0.11	0.35	0.35	0.31	0.18	0.37	0.17	0.33	0.11
PAOX	cg17143376	0.80	0.69	0.41	0.35	0.72	0.38	0.15	0.26	0.22	0.08	0.04	0.15	0.12	0.06
HOXB3	cg06186155	0.24	0.44	0.75	0.84	0.70	0.84	0.89	0.96	0.96	0.87	0.95	0.95	0.90	0.88
MEIS1	cg11357542	0.27	0.39	0.66	0.75	0.62	0.73	0.91	0.90	0.87	0.91	0.90	0.89	0.90	0.90
HLF	cg08865625	0.11	0.28	0.73	0.80	0.57	0.84	0.87	0.89	0.86	0.85	0.88	0.88	0.87	0.87
FAM159A	cg12527995	0.76	0.64	0.34	0.08	0.28	0.43	0.07	0.06	0.07	0.07	0.06	0.06	0.07	0.06
STK17A	cg17707057	0.82	0.72	0.59	0.37	0.75	0.61	0.07	0.12	0.10	0.09	0.11	0.11	0.08	0.06
IL10RA	cg05468843	0.81	0.72	0.45	0.24	0.60	0.60	0.05	0.04	0.04	0.05	0.40	0.07	0.10	0.19
CUX1	cg14203426	0.27	0.23	0.27	0.89	0.86	0.12	0.91	0.99	0.98	0.95	0.99	0.98	0.87	0.64
TESC	cg06768361	0.24	0.19	0.16	0.92	0.85	0.04	0.97	0.98	0.98	0.95	0.98	0.97	0.89	0.57
UBE2V1	cg11390541	0.17	0.27	0.28	0.52	0.31	0.21	0.76	0.82	0.84	0.76	0.85	0.80	0.71	0.57
MYO1D	cg00164282	0.79	0.61	0.64	0.54	0.70	0.77	0.07	0.06	0.06	0.08	0.08	0.06	0.06	0.05
SP140	cg17607231	0.86	0.73	0.86	0.85	0.87	0.87	0.15	0.18	0.30	0.11	0.08	0.19	0.19	0.19
CD48	cg13311440	0.74	0.63	0.67	0.59	0.68	0.76	0.18	0.09	0.11	0.26	0.18	0.15	0.19	0.19

The values of 6 out of these 18 CpGs were used as reference matrix in the NNLS models.

Supplemental Table S5. Table with calculation of mean beta-values and variances for selection of candidate CpGs.

This table provides normalized beta-values for individual CpGs that were used to calculate the difference of the mean values of both corresponding groups, and the sum of their variances. The table provides only CpGs with a mean difference >0.4 or <-0.4. Please note, that selection of CpGs was also based on the sum of variances. We arbitrarily selected the most relevant CpGs according to both parameters, taking corresponding gene functions into account as well.

Supplemental Table S6. Application for NNLS-model with 6 CpGs

An application for HSPC-subset deconvolution based on 6 CpGs is provided as separate Excel tool. This table was generated in analogy to the NNLS application for Epi-Blood-Count (3).

Supplementary References

1. Aranyi T, Stockholm D, Yao R, Poinsignon C, Wiart T, Corre G, et al. Systemic epigenetic response to recombinant lentiviral vectors independent of proviral integration. Epigenetics Chromatin. 2016;9:29.
2. Reinius LE, Acevedo N, Joerink M, Pershagen G, Dahlen SE, Greco D, et al. Differential DNA methylation in purified human blood cells: implications for cell lineage and studies on disease susceptibility. PLoS ONE. 2012;7(7):e41361.
3. Frobel J, Bozic T, Lenz M, Uciechowski P, Han Y, Herwartz R, et al. Leukocyte Counts Based on DNA Methylation at Individual Cytosines. Clin Chem. 2018;64(3):566-75.
4. Neuberger EWI, Sontag S, Brahmer A, Philippi KFA, Radsak MP, Wagner W, et al. Physical activity specifically evokes release of cell-free DNA from granulocytes thereby affecting liquid biopsy. BioRxiv. 2021;Preprint.
5. Jung N, Dai B, Gentles AJ, Majeti R, Feinberg AP. An LSC epigenetic signature is largely mutation independent and implicates the HOXA cluster in AML pathogenesis. Nature Communications. 2015;6(1):8489.
6. Aryee MJ, Jaffe AE, Corrada-Bravo H, Ladd-Acosta C, Feinberg AP, Hansen KD, et al. Minfi: a flexible and comprehensive Bioconductor package for the analysis of Infinium DNA methylation microarrays. Bioinformatics. 2014;30(10):1363-9.
7. Fortin JP, Triche TJ, Jr., Hansen KD. Preprocessing, normalization and integration of the Illumina HumanMethylationEPIC array with minfi. Bioinformatics. 2017;33(4):558-60.
8. Maie T, Schmidt M, Erz M, Wagner W, Costa IG. CimpleG: Finding simple CpG methylation signatures. BioRxiv. 2022;507513.
9. Babicki S, Arndt D, Marcu A, Liang Y, Grant JR, Maciejewski A, et al. Heatmapper: web-enabled heat mapping for all. Nucleic Acids Res. 2016;44(W1):W147-53.
10. Novershtern N, Subramanian A, Lawton LN, Mak RH, Haining WN, McConkey ME, et al. Densely interconnected transcriptional circuits control cell states in human hematopoiesis. Cell. 2011;144(2):296-309.
11. Ritchie ME, Phipson B, Wu D, Hu Y, Law CW, Shi W, et al. limma powers differential expression analyses for RNA-sequencing and microarray studies. Nucleic Acids Res. 2015;43:e47.
12. Broxmeyer HE, Srour EF, Hangoc G, Cooper S, Anderson SA, Bodine DM. High-efficiency recovery of functional hematopoietic progenitor and stem cells from human cord blood cryopreserved for 15 years. Proc Natl Acad Sci U S A. 2003;100(2):645-50.
